# Diboronic-Acid-Based Electrochemical Sensor for Enzyme-Free Selective and Sensitive Glucose Detection

**DOI:** 10.3390/bios13020248

**Published:** 2023-02-09

**Authors:** Joong-Hyun Kim, Hongsik Choi, Chul-Soon Park, Heung-Seop Yim, Dongguk Kim, Sungmin Lee, Yeonkeong Lee

**Affiliations:** 1 Drug Manufacturing Center, Daegu-Gyeongbuk Medical Innovation Foundation, 80, Chumbok-ro, Dong-Gu, Daegu 41061, Republic of Korea; 2 Medical Device Development Center, Daegu-Gyeongbuk Medical Innovation Foundation, 80, Chumbok-ro, Dong-gu, Daegu 41061, Republic of Korea; 3 Department of Biomedical Engineering, Chungbuk National University, 1, Chungdae-ro, Seowon-gu, Cheongju 28644, Republic of Korea

**Keywords:** diabetes, glucose monitoring, boronic acid, electrochemical sensor, self-assembly

## Abstract

A diboronic acid anthracene-based fluorescent system for detecting blood glucose could be used for 180 days. However, there has not yet been a boronic acid immobilized electrode to selectively detect glucose in a signal-increased way. Considering malfunctions of sensors at high sugar levels, the electrochemical signal should be increased proportionally to the glucose concentration. Therefore, we synthesized a new diboronic acid derivative and fabricated the derivative-immobilized electrodes for the selective detection of glucose. We performed cyclic voltammetry and electrochemical impedance spectroscopy with an Fe(CN)_6_^3−/4−^ redox pair for detecting glucose in the range of 0–500 mg/dL. The analysis revealed increased electron-transfer kinetics such as increased peak current and decreased semicircle radius of Nyquist plots as the glucose concentration increased. The cyclic voltammetry and impedance spectroscopy showed that the linear detection range of glucose was 40 to 500 mg/dL with limits of detection of 31.2 mg/dL and 21.5 mg/dL, respectively. We applied the fabricated electrode to detect glucose in artificial sweat and obtained 90% of the performance of the electrodes in PBS. Cyclic voltammetry measurements of other sugars such as galactose, fructose, and mannitol also showed linear increased peak currents proportional to the concentrations of the tested sugars. However, the slopes of the sugars were lower than that of glucose, indicating selectivity for glucose. These results proved the newly synthesized diboronic acid is a promising synthetic receptor for developing a long-term usable electrochemical sensor system.

## 1. Introduction

Approximately 537 million people aged 20–79 years worldwide suffer from diabetes, and that number is increasingly growing [1]. By 2045, the total number of people with diabetes will be 783 million worldwide. Although blood glucose monitoring is the gold standard for treating diabetes, sampling capillary blood five times daily is painful and increases patient noncompliance. Because patients who have insulin-dependent or Type I diabetes are vulnerable to hypoglycemia, which could lead to diabetic coma or death if their blood glucose level is not properly controlled using insulin or other drugs, blood glucose levels must be monitored often [2]. Because blood glucose levels vary individually and fluctuate daily, finger blood sampling limits the effective treatment of diabetic patients. Therefore, continuous glucose monitoring (CGM) is recommended. Currently, only four CGM systems, such as the Abbott Freestyle Libre 2, the Dexcom G6, the Guardian™ Connect System, and the Eversense CGM System, are commercially available [3]. Although these CGM systems monitor blood glucose levels accurately, few patients have benefited from them because replacing system sensors is inconvenient and maintaining these systems is expensive.

Tremendous efforts have been made to overcome the limitations of current glucose monitoring systems. For instance, tattoo-like electrochemical sensors were developed using a flexible glucose-oxidase-modified transducer for iontophoretically extracting interstitial fluid and noninvasively detecting blood glucose levels [4]. Additionally, a sweat-collecting wearable microfluidic device was integrated with a glucose-oxidase-immobilized electrode for detecting blood glucose levels [5]. Furthermore, a glucose-sensor-embedded contact lens was used for detecting glucose in tears [6]. Recently, microneedle arrays have attracted considerable research interest as an alternative method for noninvasively sampling blood glucose [7]. However, because these alternative methods rely on enzymes for selectively and sensitively detecting glucose, the short-term lifetime due to the instability of the enzymes must still be overcome. These enzyme-derived disadvantages have drawn much attention to the catalytic oxidation of glucose on electrodes consisting of metals, metal alloys, polymers, carbon nanotubes, and graphene [8]. Although showing promise for the sensitive and selective detection of glucose, these methods face challenges in overcoming the stability problem and the low poison tolerance. Moreover, the sampling-free optical methods using thermal emission, photoacoustic spectroscopy, near-infrared (NIR) and mid-infrared (MIR) spectroscopy, optical polarimetry, and Raman spectroscopy, still require more effort to miniaturize devices for patient self-use [9].

Among commercially available CGM systems, one implantable Eversense CGM System alone enables continuous glucose monitoring for up to 180 days [9]. The system relies on the increased fluorescence of a boronic acid–anthracene derivative when a boronic acid–glucose complex is formed [10]. Particularly, diboronic acid derivatives have been extensively applied to glucose detection because of their selective, fast, and strong binding with glucose reversibility. Because the synthetic receptor is thermally stable and easily protected from oxidation by attaching electron-withdrawing molecules such as trifluoromethyl, or covering it with reactive oxygen species oxidizing metal layer such as Pt, the diboronic acids can be used for a long time [10,11]. However, the other three CGM systems relying on glucose-dependent enzymes on electrodes can only be worn for up to 14 days. In addition to their potential long-term stability, the easy tailorability of the organic small receptors with substations of various functional groups has allowed several detecting formats such as UV-vis absorption, fluorescence, plasmonics, and SPR [12]. In addition to such formats, boronic-acid-based electrochemical glucose sensors should be developed because of their robustness, easy miniaturization, excellent detection limits, and small sampling volumes. In several studies, boronic-acid-derivative-based electrodes were used for electrochemically detecting saccharides [13]. Although these studies showed promising results, the electrodes did not selectively detect glucose because the single boronic acid molecules broadly bound to other 6-carbon sugars. In contrast, diboronic acids and *bis*-boronic acid interact with glucose diols more strongly than with other sugars. For example, Wang et al. reported a decreased peak current of a *bis*-boronic-acid-modified electrode for detecting glucose as low as 1 µM [14]. However, the detection range was below the range required by the FDA [15]. An electrochemical signal should be increased proportionally to the blood sugar levels, otherwise sensor malfunctions can be misinterpreted as high blood sugar levels. There was one report which showed an increase in the potentiometric signal with glucose [16]. Because diboronic acids need to be extracted from samples to polymeric liquid membranes to change the phase boundary potential, this format of the method is only suitable for in vitro applications. Recently, we synthesized a diboronic acid–anthracene derivative and demonstrated that it selectively detected glucose with high sensitivity [17]. For this study, we synthesized a new diboronic acid derivative with two primary amine groups and applied the derivative to selectively increase the electrochemical signal for detecting glucose. We immobilized the diboronic acid derivative on a screen-printed gold electrode (SPGE) surface and investigated the electrode performance for electrochemically detecting various 6-carbon sugars such as glucose, galactose, fructose, and mannitol by cyclic voltammetry (CV) and electrochemical impedance spectroscopy (EIS) with a 1:1 Fe(CN)_6_ ^3−/4−^ redox pair. The peak current increased and the semicircle radius of Nyquist plots decreased with increasing reacted glucose content, indicating faster electron transfer between the redox couple and the electrode. Moreover, the peak current increased linearly for glucose in the range of 40–500 mg/dL, which is within the United States Food and Drug Administration (USFDA)-prescribed glucose detection range [15]. For a clinical application, we devised a glucose detection method without adding the redox pair to the sample by attaching to the electrode an asymmetric membrane within which the redox pair was preloaded and dried. Additionally, the attached membrane reduced the sample volume by 2.5-fold.

## 2. Materials and Methods

### 2.1. Materials

For the sugars and the synthesis of the boronic acid derivative, the same materials were used as in our previous report [17].

10 × PBS (1440 mg/dL KH_2_PO_4_, 9000 mg/dL NaCl, 4210 mg/dL Na_2_HPO_4_, pH 7.4) was purchased from GenDEPOT (Katy, TX, USA). Artificial human sweat was purchased from Biochemazone (Alrta, Canada). Screen-printed gold electrode (220BT) was purchased from Metrohm DropSens (Austrias, Spain). N-Ethyl-N′-(3-dimethylaminopropyl) carbodiimide hydrochloride (EDC), N-Hydroxysuccinimide (NHS), K_3_[Fe(CN)_6_], and K_4_Fe(CN)6 · 3H_2_O were purchased from Sigma-Aldrich (St. Louis, MO, USA). H_2_SO_4_ (95%) was purchased from Daejung Chemicals & Metals (Gyeonggi-do, Republic of Korea). All chemicals were used as received without any further purification unless otherwise specified.

### 2.2. Methods

#### 2.2.1. Synthesis of a Diboronic Acid Derivative

**Scheme 1:**
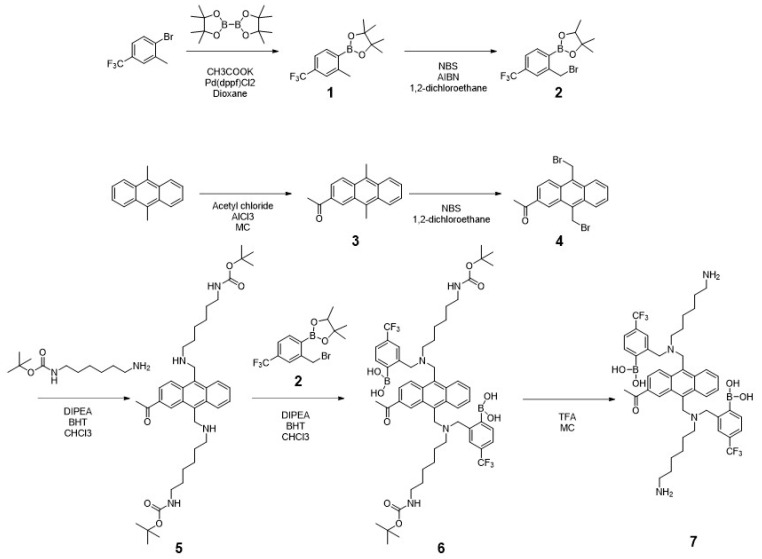
Seven steps required for synthesizing diboronic anthracene with two primary amines for electrochemically detecting glucose.

An acetylated diboronic anthracene with two primary amines was prepared according to Scheme 1. The detailed synthesis procedures of Compound 4 from the starting materials were described in our previous study [17]. The other synthesis procedures are described as follows.

#### 2.2.2. Synthesis of di-tert-butyl ((((2-acetylanthracene-9,10-diyl) bis(methylene)) bis (azanediyl )) bis(hexane-6,1-diyl))dicarbamate (Compound 5)

A total of 0.012 mol of Compound 4, 0.123 mol of NN-diisopropylethylamine (DIPEA), 0.062 mol of tert-butyl (6-aminohexyl) carbamate, and 0.001 mol of butylated hydroxy-toluene (BHT) were dissolved in 150 mL at 30 °C for 18 h. After evaporating the solvent, the mixture was washed with 200 mL of distilled water (D.W) and the D.W washing was repeated twice more and then the mixture was dried over MgSO_4_. The obtained crude product was eluted through silica gel column chromatography using dichloromethane/MeOH (9:1, *v*/*v*). A yellow powder was obtained. Yield: (5.3 g, 63.6%). ¹H NMR (400 MHz, Chloroform-d): δ 9.10-7.56(m, 7H), 4.77(s, 2H), 4.69(s, 2H), 4.64-4.52(m, 2H), 3.44-2.53(m, 11H), 1.61-1.35(s, 36H). LC MS: Calcd. for C_40_H_60_N_4_O_5_ *m*/*z*: 676.94; found *m*/*z*: 677 [M+H]^+^.

#### 2.2.3. Synthesis of (((((2-acetylanthracene-9,10-diyl) bis(methylene)) bis((6-((tert-butoxycarbonyl) amino) hexyl) azanediyl)) bis(methylene)) bis(4-(trifluoromethyl)-2,1-phenylene)) diboronic acid (Compound 6)

A total of 0.0078 mol of Compound 5 and 0.156 mol of DIPEA (27.3 mL) was dissolved in 20 mL of chloroform and the mixture was stirred at RT. A total of 0.001 mol of BHT and 0.039 mol of 2-(2-(bromomethyl)-4-(trifluoromethyl)phenyl)-4,4,5,5-tetramethyl-1 and 3,2-dioxoborolane) was added to the mixture, and stirred at RT for at least 24 h. The solvent was removed under reduced pressure. After dissolving the mixture in 20 mL isopropyl ether, it was washed 3 times with 20 mL of phosphate buffer (0.2 M, pH 7). After removing the solvent, the mixture was eluted through silica gel column chromatography with dichloromethane/MeOH (98:2, *v*/*v*) as the eluent. A total of 12.0 g of yellow powder was obtained in a yield of 141.8%. ¹H NMR (400 MHz, Chloroform-d): δ 9.12-7.58(m, 13H), 4.80(s, 2H), 4.72(m, 3H), 4.66(s, 2H), 4.58(m, 3H), 3.44-2.53(m, 15H), 1.61-1.35(m, 34H). LC-MS: Calcd. for C_56_H_72_B_2_F_6_N_4_O_9_ *m*/*z*: 1080.82; found *m*/*z*: 1081 [M+H] ^+^; 1063 [M-H_2_O+H]^+^, 1045 [M-2H_2_O+H]^+^.

#### 2.2.4. Synthesis of (((((2-acetylanthracene-9,10-diyl) bis(methylene)) bis((6-aminohexyl) azaned -iyl )) bis(methylene))bis(4-(trifluoromethyl)-2,1-phenylene))diboronic acid (Compound 7)

A total of 0.005 mol of Compound 6 and 20 wt% tri-fluoroacetic acid in 5 mL of methylene chloride was stirred at RT for 20 h. The solvent was evaporated under a vacuum. The product was eluted through silica gel column chromatography with CHCl_3_/MeOH (95:5, *v*/*v*). A total of 0.074 g of yellow powder was obtained in a yield of 43.5%. ¹H NMR (400 MHz, Chloroform-d): δ 9.21-7.51(m, 13H), 4.82(s, 2H), 4.74(s, 2H), 4.68(s, 2H), 4.60(s, 2H), 3.51-2.49(m, 15H), 1.55-1.30(m, 20H). LC-MS: Calcd. for C_46_H_56_B_2_F_6_N_4_O_5_ *m*/*z*: 880.59; found *m*/*z*: 881 [M+H]^+^; 863 [M-H_2_O+H]^+^, 845[M-2H_2_O+H]^+^.

### 2.3. Fabrication of Diboronic-Acid-Derivative-Immobilized Electrode

First, the electrodes were cleaned by rinsing them with ethanol (EtOH) or dropping 0.5 M H_2_SO_4_ onto them and then voltammetrically cycling them 20 times from 0 to 1.2 V with a scan rate of 20 mV/s. The cleaned electrodes were immersed for 10 min in 1 mM mercaptobenzoic acid (MBA) dissolved in EtOH. The MBA-treated electrodes were successively washed with EtOH and deionized (DI) water and dried with N_2_ gas. To conjugate the diboronic acid (DA; Compound 7) with the MBA-treated electrodes, 2 mM DA dissolved in 0.1 × PBS solution was mixed with 150 mM N-hydroxysuccinimide (NHS) and 100 mM 1-ethyl-3-[3-dimethylaminopropyl]-carbodiimide hydrochloride (EDC). Then, 20 µL of the mixture was immediately dropped onto the working electrode, which was incubated for 1 h, washed with DI water to remove any residual unreacted substances, dried with N_2_ gas, and stored under a vacuum in the dark until use.

### 2.4. Electrochemical Measurement of Fabricated Electrodes

CV measurements were performed using a portable potentiostat (uStat-i-4000; Metrohm AG, Ionenstrasse, Switzerland) and 50 µL of 10 mM Fe(CN)_6_^3−/4−^ in 0.1 × PBS containing 0.1 M KCl scanning from −0.3 V to 0.6 V at a scan rate of 50 mV/s. The redox pair was prepared by dissolving K_3_[Fe(CN)_6_], K_4_[Fe(CN)_6_] · 3H_2_O and KCl in 0.1 × PBS just before use. An amount of 0.1 × PBS was prepared by diluting 1 part 10 × PBS with 9 parts ultra-pure water. EIS measurements were carried out using the same instrument and 0.1 × PBS containing the redox pair and KCl. EI spectra were obtained in the range of 10^−1^ to 10^5^ Hz at the half-wave potential of 0.20 V with 10 mV sine wave potential.

For the electrochemical detection of sugars, 50 µL of sugars dissolved in 0.1 × PBS containing 10 mM Fe(CN)_6_^3−/4−^ and 0.1 M KCl were dropped on the electrode. After incubation for 10 min, CV or EIS measurements were carried out under the same conditions. For real sample mimicking tests, 20 µL of glucose dissolved in the artificial sweat was dropped on the electrode. After incubation for 10 min, CV measurements were carried out.

## 3. Results and Discussion

### 3.1. Electrochemical Analysis of DA-Immobilized Electrodes

In our previous report, we presented the acetylated diboronic anthracene derivative, which exhibited the highest Stokes shift and stability among the other derivatives. Moreover, the derivative exhibited excellent sensitive and selective glucose detection [17]. To immobilize the diboronic anthracene derivative on the working electrode surface, a water-based buffer is preferable because organic solutions spread electrodes, including reference and counter ones, thereby hindering selective compound immobilization. Therefore, we added two amine moieties to the acetylated diboronic anthracene derivative, because the amine molecule forms a hydrogen bond with water, thereby allowing high solubility of the derivative in the aqueous buffer and amide bonds with carboxylic acids for the immobilization on the electrodes.

**Figure 1:**
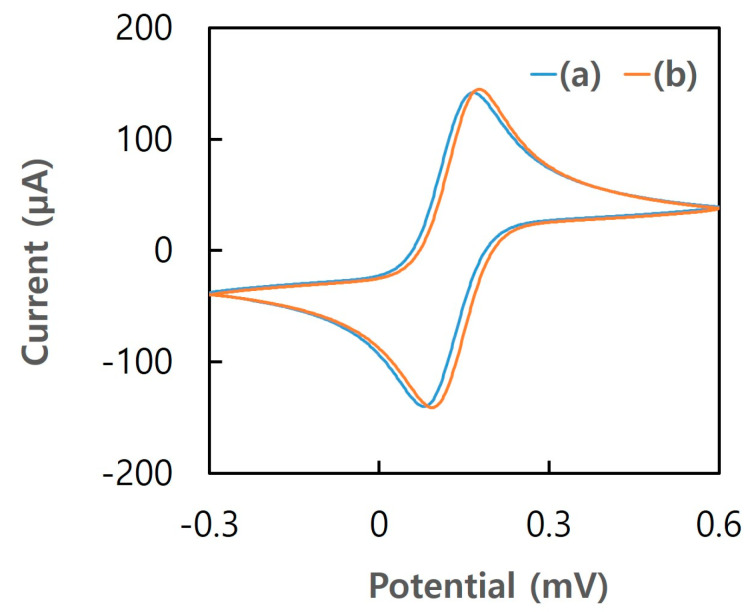
Cyclic voltammograms of electrodes cleaned by (**a**) rinsing with EtOH and (**b**) electrochemical cycling in diluted H_2_SO_4_. A total of 50 µL of 0.1 × PBS (pH 7.4) containing 10 mM Fe(CN)_6_^3−/4−^ and 0.1 M KCl was applied to the electrodes and CV measurements were carried out.

Before immobilizing the DA on the electrode surface, we investigated whether the SPGE had to be cleaned using H_2_SO_4_ because the electron transfer rate is affected by SPGE surface contamination and oxidation [18,19]. To remove any surface contamination/oxidation, the SPGE was routinely cleaned using CV at 20 mV/s between 0 and 1.2 V by immersing the SPGE in 0.5 M H_2_SO_4_ [18,19] or by simply rinsing the SPGE with EtOH. Figure 1 shows the cyclic voltammograms obtained for both cleaned SPGEs. Clearly, the difference between the voltammograms obtained for the SPGEs electrochemically cleaned in H_2_SO_4_ and those simply rinsed with EtOH was negligible. Both voltammograms exhibited almost identical peak currents and peak-to-peak separations, which are the potential differences between oxidation and reduction peak potentials and are key electron-transfer-rate indicators. Cleaner surfaces provide faster electron transfer and smaller peak separations [18,19]. Because both SPGEs exhibited the same peak separation, simply rinsing the bare SPGE with ethanol cleaned the surface as well as the electrochemical H_2_SO_4_ surface cleaning. Therefore, we only cleaned the SPGEs with ethanol. Because the SPGE contamination and oxidation both depend on the storage time and air quality, all the SPGEs were stored in a highly clean vacuum container and used within one month of fabrication.

**Scheme 2:**
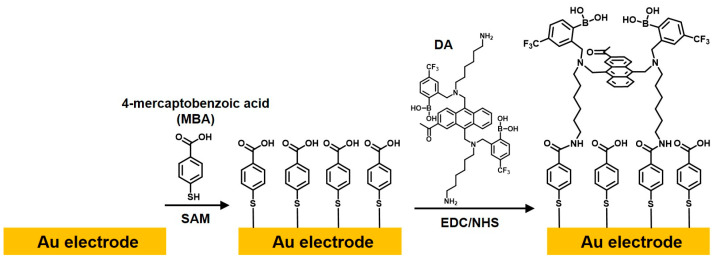
Schematics illustrating two steps required for fabricating DA-immobilized electrodes.

**Figure 2:**
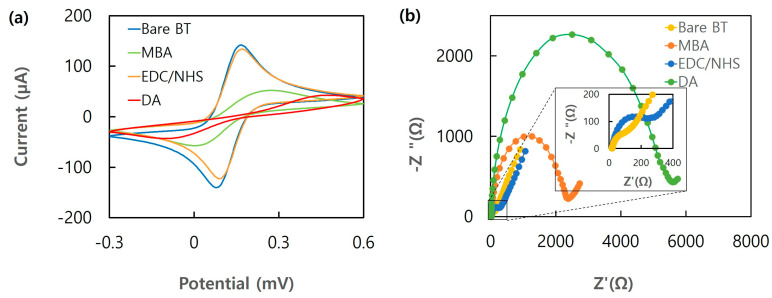
Electrochemical analysis for MBA- and DA-immobilized electrodes. (**a**) Cyclic voltammograms and (**b**) Nyquist plots obtained for surface-modified electrodes by applying 50 µL of 0.1 × PBS (pH 7.4) containing 10 mM Fe(CN)_6_^3−/4−^ and 0.1 M KCl to the electrodes. Inset shows the enlarged image of the Nyquist plots of the bare electrode and EDC/NHS treated electrode.

To fabricate a glucose-selective layer, an MBA linker layer was first self-assembled on the electrode surface and DA was then conjugated with the MBA layer (Scheme 2). For the first surface modification, ethanol-cleaned SPGEs were incubated with 1 mM MBA for 10 min and then coupled with 2 mM DA in excess EDC/NHS. Each surface modification was monitored using CV and EIS. CV is not only used as an amperometric tool for the detection of glucose but also as a useful tool to characterize the modification of the electrodes [13,14,19]. As shown in Figure 2a, the peak current and current separation decreased and increased, respectively, after the immobilization of MBA. The changes in the peak current and peak potential separation were derived from the decreased electron-transfer kinetics after the surface modification. The electrostatic repulsion between the negatively charged MBA layer and Fe(CN)_6_^3−/4−^ redox pair should increase the electron-transfer resistance on the MBA-modified surface of the electrode. Additionally, the larger immobilized layer acts as a barrier against interfacial electron transfer between the redox pair and the electrodes. As a result, there were dramatic changes in the CV curves. On the other hand, after EDC/NHS treatment of immobilized MBA, the peak current reached almost 97% of the peak current of the bare electrode. The addition of EDC/NHS replaced the negatively charged carboxyl group of MBA with neutral-charge NHS ester, which eliminated the electrostatic barrier. As a result, the peak current could increase. Then, DA-immobilized electrodes showed the lowest oxidation and reduction potentials and the highest peak separation.

The semicircle diameters in Nyquist plots also revealed consistent results with the CV analysis (Figure 2b). In EIS, the semicircle diameter represents the electron-transfer resistance, R_ct_. Because the electron-transfer kinetics of redox couple is controlled by R_ct_ at the electrode interface [14,20], EIS is an effective method for verifying the formation of each functional layer after surface modification. The appearance of a larger semicircle for the electrode after the MBA modification than that of the bare electrode indicates increased electron transfer resistance and thus confirms the electrical barrier derived from the increased size and the electrostatic repulsion as explained in the CV analysis. The dramatically decreased diameter of the semicircle after successive EDC/NHS treatment also supports the elimination of the electrostatic barrier. The DA conjugation on the MBA-modified electrode showed the largest semicircle compared to the other electrodes. The largest semicircle reflects the formation of an additional insulating layer on the electrode, which acted as a barrier to interfacial electron transfer. Therefore, the DA-conjugated electrodes exhibited the lowest peak current.

### 3.2. Electrochemical Detection of Glucose

**Figure 3:**
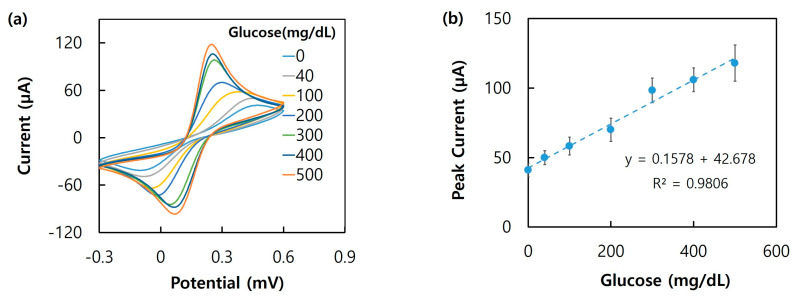
Electrochemical detection of glucose using DA-modified electrode. (**a**) CVs and (**b**) peak currents obtained after applying 50 µL of 0.1 × PBS (pH 7.4) containing 10 mM Fe(CN)_6_^3−/4−^, 0.1 M KCl, and glucose in the range of 0–500 mg/dL to the electrodes.

**Figure 4:**
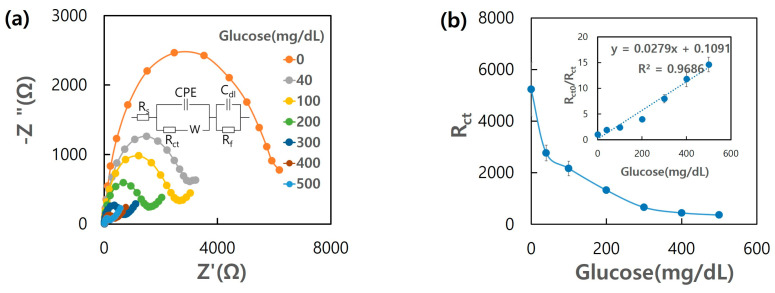
(**a**) Nyquist plots and (**b**) electron transfer resistances obtained applying 50 µL of 0.1 × PBS (pH 7.4) containing 10 mM Fe(CN)_6_^3−/4−^, 0.1 M KCl, and glucose in the range of 0–500 mg/dL to the DA-modified electrode. Inset in (**a**,**b**) is the equivalent circuit model and the relative electron transfer resistance respectively. R_cto_ represents the electron transfer resistance without glucose.

We then investigated the electrochemical glucose-detecting ability of the DA-immobilized electrode using CV and EIS measurements at various concentrations of glucose. Figure 3 and Figure 4 show the obtained voltammograms and Nyquist plots, respectively, wherein the peak current changes and semicircle diameters depended on the glucose concentration.

As shown in Figure 3a, the peak current and separation increased and decreased, respectively. The peak current was linearly correlated with increasing glucose concentration, as shown in Figure 3b. The linear glucose detection range was 40–500 mg/dL. The limit of the detection of glucose can be decided by Equation (1).

LOD = 3σ/S,(1)

where σ and S are the standard deviation of the blank and the slope obtained from the linear fitting, respectively; σ was calculated as 4.92. Then, the calculated LOD of glucose was 31.2 mg/dL.

These electrical responses of the DA-immobilized electrodes were very interesting because previous studies have reported opposite results [13,14]. Because the electron transfer slows with increasing peak separation, this decreased peak separation suggests that the electron transfer accelerated. The complex formed between DA molecules and glucose diols generates a physical barrier, which further limited redox-pair access to the electrode surface. Therefore, the peak current and separation should have decreased and increased, respectively, after glucose was added to the electrode. Instead, the opposite observation indicates accelerated electron transfer or decreased electron resistance between the redox pair and electrode surface. Compared to previous studies, this study used two phenyl boronic acid molecules with a central anthracene molecule for interacting with glucose.

**Figure 5:**
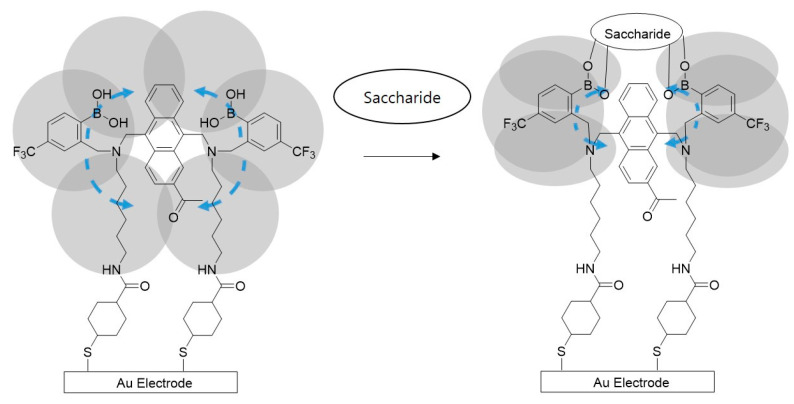
Simplified illustration to depict space occupied by moving phenyl rings depending on interacting with saccharide molecules. The gray area indicates space generated by rotating or vibrating phenyl rings. Freely moving phenyl rings should occupy more space than saccharide-linked ones.

As shown in Figure 5, before the complex is formed, the freely moving phenyl rings should hinder redox-pair access to the electrode. In contrast, when the complex is formed, both boronic acid molecules are connected through a glucose link between both phenyl groups [21]. Then, the formation of the boronic-acid–glucose complex should suppress the free rotation or vibration of the phenyl rings and thus the two-phenyl-group connection can generate additional room, thereby enabling the redox pair to access the surface easily. Instead, the complex formed between the glucose and single boronic acid molecules only increased the physical size of the barrier against the redox-pair movement to the electrode rather than restricting the immobilized layer thermal motions.

Another difference in the boronic acid derivative used in this study was that the DA was immobilized on the electrodes with two carbon chains. Reportedly, movement of molecules attached to electrodes only at a single point was more flexible than that of molecules attached to electrodes at two points [22]. Although the *bis*-boronic-acid-modified electrodes showed selectivity for glucose molecules, the peak current and separation decreased and increased, respectively. Because *bis*-boronic acid molecules were attached to the electrode surface through a single carbon chain, the overall thermal motion of the immobilized complex was hardly affected after the glucose molecule was bound to the boron atom. Therefore, the overall thermal motion of the immobilized complex negligibly influenced the interfacial electron transfer. Instead, the larger glucose-bound immobilized *bis*-boronic acid layer hindered the interfacial electron transfer. Moreover, because glucose-bound boron is negatively charged [21,23,24], the doubly negatively charged *bis*-boron repelled [Fe(CN)_6_^3−/4−^] anions. The electrostatic repulsion between the negatively charged boron and redox pair should increase the electron-transfer resistance on the surface and thus result in the negative changes of the signal. In this work, the diboronic acid–anthracene had two nitrogen that controlled the electrical charge of the boron. According to the PET mechanism, the glucose-bound boron anion of the diboronic acid–anthracene derivative inhibited interactions between the positively charged nitrogen and anthracene and enabled boron (Lewis acid)–nitrogen (Lewis base) interaction [21,23,24]. The electrostatic repulsion between the boron and redox couple disappeared for the DA-immobilized electrode because the anthracene–nitrogen electrostatic interaction switched to the boron–nitrogen one. The crucial role of the electrostatic interaction between the redox pair and the surface of the electrode was already confirmed with the CV and EIS analysis of the EDC/NHS-treated electrode. As a result, the peak current of the electrodes with glucose could reach 80% of the peak current of the bare electrode.

The decreased electron-transfer resistance was analyzed using EIS measurement. Figure 4 shows the Nyquist plots obtained from the EIS measurement for the DA-immobilized electrode at different glucose concentrations. With increasing glucose concentration, the semicircle diameter decreased, revealing that the electron-transfer resistance decreased proportionally to the glucose concentration. A circuit (inset in Figure 5b) was used to fit the measured data. In the circuit, R_st_ is the solution resistance that is related to the conductivity/mobility of the redox probe in solution, R_ct_ is the electron transfer resistance, W is Warburg impedance representing semi-infinite linear diffusion within the solution, and CPE is the constant phase element. C_dl_ and R_f_ represent the double layer capacitance and the resistance at the immobilized layer/electrode interface, respectively. Since the redox probe should face the immobilized layer to exchange electrons, the accessibility of the redox probes is affected by target–receptor interactions at the immobilized receptor/electrode interface. Therefore, R_ct_ has been commonly used to quantify the barrier the redox probes faced [14,25,26,27,28]. As shown in Figure 5b, R_ct_ decreased with the increasing tested concentration of glucose. The decrease in the electron transfer resistance means an increase in the accessibility of the redox pair to the electrode. The EIS and CV measurements agreed well, which confirmed that the decreased electron-transfer resistance over the changed glucose-bound DA structure increased the peak current and decreased the current separation proportionally to the glucose concentration. The relative R_ct_ is plotted as a function of the glucose concentration (Figure 5b inset). Clearly, the electrical resistance was linearly correlated with the glucose concentration in the range of 40–500 mg/dL. The σ for the LOD of glucose in the EIS measurements was 0.20. Then, the LOD of glucose in the EIS measurement was decided at 21.5 mg/dL, which was slight lower than the LOD in CV measurements. Wang et al. also reported a slightly higher binding constant for glucose in EIS measurements than that in CV measurements [14].

To detect the glucose concentration in a real sample, the redox-pair-adding step needed to be eliminated. An asymmetric membrane through which fluid moved in only one direction was attached to the DA-immobilized electrode surface. A concentrated redox-pair solution was dropped and dried on the membrane surface before the attachment. Because the membrane was attached to the electrode surface such that sample drops penetrated the electrode, most of the glucose in the sample and redox pair interacted with the electrode surface. Additionally, because water drops did not form but instead spread out evenly on the membrane surface, the attached membrane enabled covering the surface of the electrode with less volume of sample (Supplementary Materials, Figure S1). Too much sample loaded on the membrane surface could submerge the membrane and, thus, eliminate the asymmetric effect. A sample loading of 20 μL was enough to wet the membrane effectively without exceeding the liquid absorption capacity limit. First, we determined the volume of the redox pair required on the membrane surface to achieve an electron transfer rate like that of the membrane-free redox pair. CV measurements were carried out after 20 μL of 0.1 × PBS were added to the membrane-attached electrodes.

**Figure 6:**
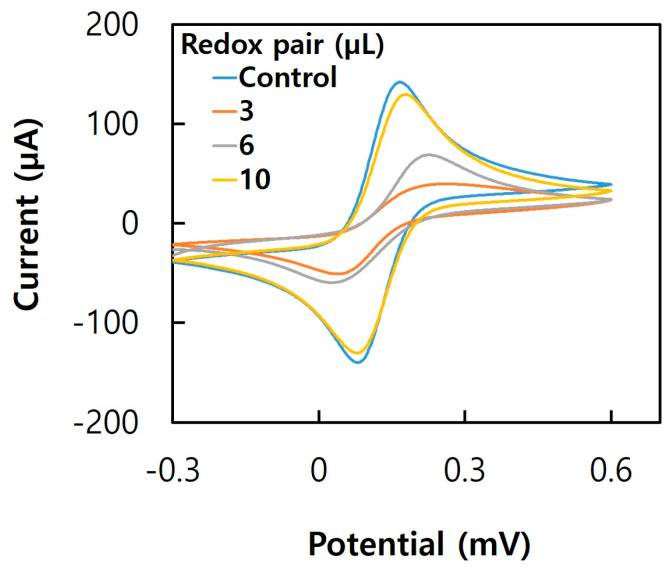
Cyclic voltammograms obtained by applying 20 µL of 0.1 × PBS (pH 7.4) to the asymmetric-membrane-attached electrodes depending on added amount of the redox pair.

Figure 6 shows the CV measurements obtained by adding different amounts of redox pair to the membrane. As shown in Figure 6, the peak current increased as the added amount of the redox pair increased. When 10 μL of the concentrated redox pair was added to the membrane, the peak current was almost identical to that of the membrane-free electrode. Then, we used the membrane to detect glucose in artificial sweat electrochemically.

**Figure 7:**
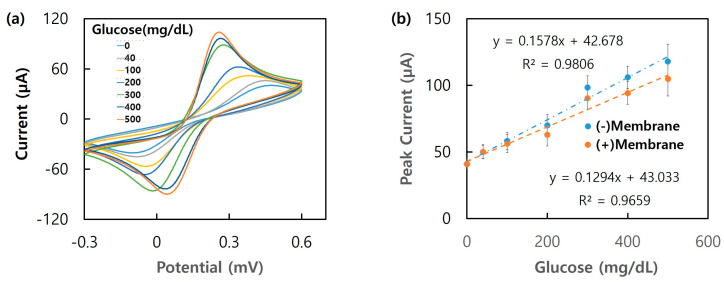
Electrochemical detection of glucose in artificial sweat using DA-modified electrode with asymmetric membrane. (**a**) Cyclic voltammograms and (**b**) peak currents obtained using glucose in the range of 0–500 mg/dL. 20 µL of artificial sweat containing glucose was applied to the electrodes and CV measurements were carried out.

We simulated glucose detection in a real sample using artificial sweat. Figure 7 compares peak currents measured using CV for the membrane-based and membrane-free electrodes for artificial sweat containing glucose in the range of 0–500 mg/dL. As shown in Figure 7b, the peak currents obtained using the membrane-based electrode linearly increased with increasing fluidic glucose concentration in the range of 0–500 mg/dL and were ~90% of those obtained using the membrane-free electrode. The LOD of glucose in sweat was calculated at 28.0 mg/dL. This result demonstrated good performance of the DA-immobilized electrodes with the membrane in the real sample. However, glucose concentration in sweat is in the range of 1.0–3.6 mg/dL (0.06–2.0 mM) [29]. In order to detect glucose in sweat, the LOD should be improved by at least a factor of 28. Due to the high surface area to volume ratio, 1-D nanostructure base field-effect transistors have shown high sensitivity [30]. Particularly, boronic acid functionalized carbon nanotube transistors have responded to glucose as low as 0.6 µg/dL [31]. Therefore, immobilization of DA to the FET transistor could allow the required LOD for the detection of glucose in sweat.

### 3.3. Selectivity for Electrochemical Detection of Glucose

Selective glucose detection is very important, especially for drug-reliant diabetes patients, because coexisting glucose-binding interfering substances can lead to false positive detections and, subsequently, wrong medication or insulin dosages [32,33]. The glucose selectivity of the DA-immobilized electrodes was investigated using glucose-similar 6-carbon sugars such as galactose, fructose, and mannitol. CV measurements were carried out using the DA-immobilized electrodes by dropping various concentrations of 6-carbon sugars onto the electrode surfaces.

**Figure 8:**
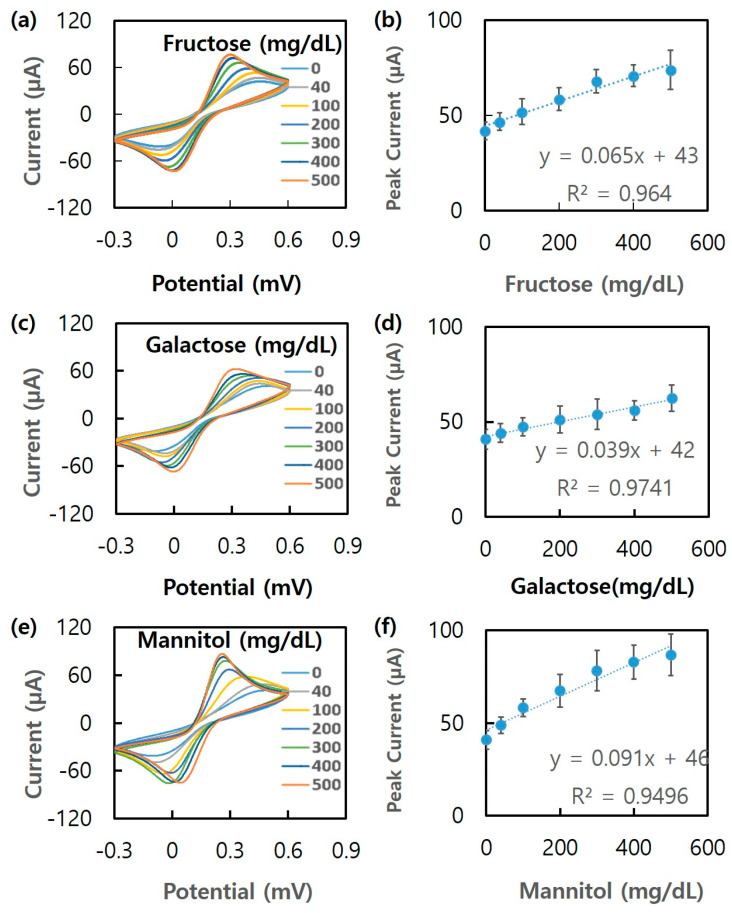
Electrochemical selectivity of DA-immobilized electrode for detecting saccharides. in the range of 0–500 mg/dL. 50 µL of 0.1 × PBS (pH 7.4) congaing 10 mM Fe(CN)_6_^3−/4−^, 0.1 M KCl, was applied to the electrodes and CV measurements were carried out (**a**,**c**,**e**) Cyclic voltammograms and (**b**,**d**,**f**) corresponding peak currents obtained using fructose, mannitol, and galactose, respectively.

Figure 8a,c,e show the cyclic voltammograms obtained using these three sugars. The peak current increased and separation decreased with increasing sugar concentration, suggesting that the sugar bound to the boronic acids molecules likely induced the formation of a structure more rigid than that of the free phenyl rings, which enabled the redox pair to approach the electrode easily and increased the electron transfer. Figure 8b,d,f compare the peak currents obtained using these three sugars. Clearly, the slopes obtained for the peak currents of all the glucose-interfering sugars were lower than that obtained for the glucose peak current, indicating that DA bound more strongly to glucose than to the other sugars. Moreover, the slope order agreed well with the association constants previously reported for the analog [14,17,21,23,24].

Because actual blood glucose concentrations are at least 36-fold higher than those of the interfering sugars, the electrochemical signal intensities originating from the other sugars would be <1.6% of the one originating from glucose [23,34,35], suggesting that the signals originating from interfering sugars were negligible. For example, the peak current with glucose is about 2.5 times higher than the peak current with fructose. Plasma fructose is 0.1% of glucose [28]. The actual peak current from fructose would be only 0.04% of the peak current from glucose. Besides the tested sugars, other saccharides such as lactose, glucosamine, and sucrose are present in body fluids. According to the work by Wang et al., binding affinities of DA analogues toward lactose, glucosamine, and sucrose were less than 2.7 × 10^−3^ of that to glucose or not detectable [23]. While still requiring further experimental proof, the saccharides would not interfere with the DA-immobilized electrodes. To our knowledge, this is the first study to report a higher increase in the electrochemical signal from DA-immobilized electrodes for glucose than for other sugars. Therefore, these results prove that DA could be a promising synthetic receptor for the electrochemical detection of glucose in the presence of other sugars.

## 4. Conclusions

DA was used to fabricate an electrochemical sensor for enzyme-free selective and sensitive glucose detection. DA-immobilized electrodes were fabricated by conjugating DA with MBA immobilized on the gold surface of SPGEs. After each surface modification, CV and EIS measurements revealed increased electrical resistance on the electrode surface, which confirmed the successful immobilization of the functional molecules on the electrodes. Like electrochemical signal changes measured using enzyme-based glucose sensors, the peak current and electrical resistance increased and decreased, respectively, with increasing glucose concentration. Positive signal changes are very important for preventing sensor malfunctions from being misinterpreted as increased blood glucose levels. The formation of a boronic acid–glucose complex locked both phenyl rings where the boronic acid molecule attached. The conformational change from freely rotating and vibrating phenyl rings to a rigid structure generated more room for the redox pair to access the gold electrode and, thus, mainly accelerated the electron transfer, which increased and decreased the peak current and surface electron resistance, respectively, proportionally to the increased glucose concentration. As a result, the LODs with the DA-immobilized electrodes were 31.2 mg/dL and 21.5 mg/dL in CV and EIS measurements, respectively. The obtained linear glucose detection ranges from 40 to 500 mg/dL satisfied the USFDA requirement for clinical applications. Additionally, the DA-immobilized electrodes enabled selective glucose detection, which is also very important for clinical applications. The asymmetric membrane enabled 90% recovery of the membrane-free electrochemical signal without adding the redox pair to the test samples and reduced the sample volume by 2.5-fold, which are both critical for clinically applying the fabricated sensor. Because the sample volume is determined by the electrode size, the volume required for detecting glucose can be easily reduced by fabricating an electrode with a size like that of the commercially available glucose sensors. This study demonstrated the first-ever use of DA-immobilized electrodes for selectively electrochemically detecting glucose by the positive changes in the signal. Therefore, we believe these results will provide fundamental insight for stimulating further research into using DA to electrochemically detect glucose more feasibly and stably and will contribute to the care of diabetes patients.

## Data Availability

Not applicable.

## Supplementary Materials

The following supporting information can be downloaded at: https://www.mdpi.com/article/10.3390/bios13020248/s1, Figure S1: Pictures showing 20 µL of sample drops on the electrode surfaces (a) without and (b) with the asymmetric membrane.
