# Supplementary material for: Diboronic-Acid-Based Electrochemical Sensor for Enzyme-Free Selective and Sensitive Glucose Detection
Source: Biosensors (Basel). 2023 Feb 9;13(2):248. doi: 10.3390/bios13020248 (PMC9954471; doi:10.3390/bios13020248)
Supplement: Supplementary file 1 [file biosensors-13-00248-s001.zip › biosensors-2082189-supplementary.pdf]

(a) (b)

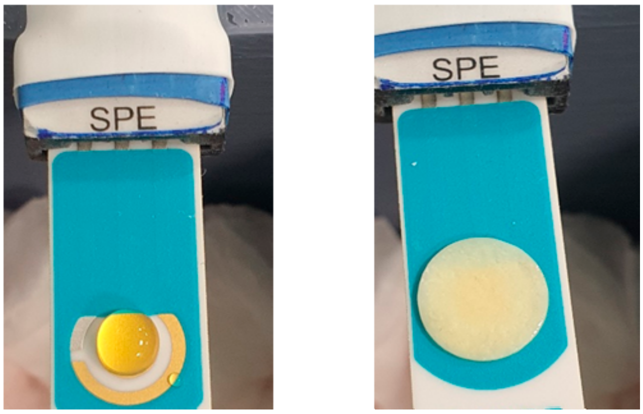

Figure S1. Pictures showing 20 μL of sample drops on the electrode surfaces (**a**) without and (**b**) with the asymmetric membrane.
